# A review and bibliometric analysis of global research on non-pharmacologic management for neonatal and infant procedural pain

**DOI:** 10.1097/MD.0000000000040552

**Published:** 2024-11-29

**Authors:** Xin Chen, Ruoyu Li, Anqi Xiong, Biru Luo

**Affiliations:** a Department of Obstetrics Nursing, West China Second University Hospital, Sichuan University/West China School of Nursing, Sichuan University, Chengdu, China; b Key Laboratory of Birth Defects and Related Diseases of Women and Children (Sichuan University), Ministry of Education, Chengdu, China; c Department of Nursing, West China Second University Hospital, Sichuan University/West China School of Nursing, Sichuan University, Chengdu, China.

**Keywords:** bibliometric, CiteSpace, neonatal pain, non-pharmacologic management, VOSviewer

## Abstract

Repeated and prolonged exposure to pain can impair neurodevelopmental, behavioral, and cognitive outcomes in newborns. Effective pain management of newborns is essential, but there is no comprehensive analysis of the status of neonatal pain non-pharmacologic management research. Original publications related to the non-pharmacological management of neonatal pain were obtained from the Web of Science Core Collection (WOSCC) between 1989 and 2024. CiteSpace and VOSviewer were used to extract information about countries/regions, institutions, authors, keywords, and references to identify and analyze the research hotspots and trends in this field. 1331 authors from 51 countries and 548 institutions published studies on the non-pharmacological management of neonatal pain between 1989 and 2024, with the number of publications showing an overall upward trend. Canada emerged as the leading country in terms of publication volume, with the University of Toronto and The Hospital for Sick Children identified as key research institutions. High-frequency keywords included “procedural pain,” “management,” “sucrose,” “analgesia,” and “preterm infant,” resulting in 11 clusters. Keyword emergence analysis revealed that “neonatal pain,” “analgesia,” “oral sucrose,” and “oral glucose” were research hotpots. Analysis of highly cited papers showed that the most referenced articles were published in the Clinical Journal of Pain. Researchers’ interest in neonatal procedural pain has increased significantly over the past 30 years. This article can serve as a theoretical reference for future research on mild to moderate pain in neonates and infants, and it can provide ideas for exploring novel and secure pain management strategies.

## 1. Introduction

Numerous Studies have demonstrated that neonates in intensive care often undergo repeated invasive procedures.^[1]^ For example, according to a systematic review, neonates in the first 14 days of life or during their stay in the intensive care unit experienced between 7.5 and 17.3 invasive medical procedures per day, with 6832 to 42,413 procedures identified.^[2]^ In addition, a cross-sectional survey conducted in Kenya in 2014 pointed out that neonates went through a total of 404 painful procedures within 24 hours. Among these, 270 were tissue-damaging while 134 were non-tissue-damaging procedures. The most common painful procedures were peripheral cannula insertion (27%) and intramuscular injections (22%).^[3]^ In the same year, a prospective observational study in the Netherlands revealed that 175 neonates admitted to the NICU underwent a collective of 21,076 pain procedures during hospitalization, with an average of about approximately 120.4 per newborn. The mean number of painful procedures per neonate daily was 11.4.^[4]^ The risk of so many invasive procedures on neonates is worrying.

Research has widely shown that neonates can feel pain.^[5,6]^ Untreated pain can lead to short-term effects, such as increased intracranial pressure and oxygen desaturation, altered responses to subsequent pain, and increased long-term risks of adverse neurodevelopmental, behavioral, and cognitive outcomes.^[7–10]^ Additionally, inadequate analgesia also has an impact on medical outcomes.^[11]^ Therefore, exploring favorable pain management strategies is integral to appropriate infant procedural pain management. The consensus published by the International Evidence-Based Group for Neonatal Pain^[12]^ emphasized that pain management must be considered an important component of the health care provided to all newborns, regardless of their gestational age or disease severity, and the Pain Study Group of the Italian Society of Neonatology^[13]^ has established guidelines for managing procedural pain in newborns. These guidelines have heightened awareness among healthcare professionals about the importance of effectively managing pain in neonates.

Many pharmacological and non-pharmacological treatments can help reduce painful procedures in newborns, but long-term medication for pain may have adverse effects like prolonged mechanical ventilation, delayed feeding, impaired brain function, insufficient social behavior, and short-term memory loss.^[14,15]^ By contrast, many non-pharmacologic therapies can help manage mild to moderate pain in newborns. Non-drug methods such as oral sucrose, kangaroo care, simulated intrauterine sound, non-nutritive sucking, skin-to-skin care, breastfeeding, and heel warming can relieve pain, reduce crying, and address behavioral issues caused by invasive procedures.^[16–21]^ For some procedural pain that cannot be managed with drugs, such as blood glucose tests and immunization, non-drug intervention can effectively relieve pain. Based on the preceding information, it is essential to concentrate on the research related to non-pharmacological interventions for neonatal procedural pain, to explore the current research progress and identify future research trends.

Bibliometric analysis is a well-established research method in information science.^[22]^ It is a discipline that conducts qualitative and quantitative analysis of the literature system and characteristics through mathematics and statistics.^[23]^ Bibliometrics can help identify research trends, emerging fields, and collaborations, and inform strategic planning within research organizations.^[24]^

Up to now, studies on non-drug analgesia for relieving neonatal procedural pain are ongoing, and these studies primarily focused on developing pain assessment methods for different target groups (preterm infants, term infants, and toddlers),^[25,26]^ as well as studying the effectiveness of various non-drug interventions for these groups,^[27,28]^ but did not specify which method is the most effective and most suitable for a certain clinical scenario. Moreover, the publication of a comprehensive review on non-pharmacological treatments for neonatal procedural pain using bibliometrics and visualization methods has not been published worldwide. To fill this knowledge gap, we conducted a study using the Web of Science Core Collection (WoSCC) to review the major contributors in this field over the past 35 years and identify research hotspots and trends, including countries, institutions, keywords and conducted co-citation analysis, etc. In Addition, we draw knowledge maps using Citespace and VOSviewer. The purpose of this study is to investigate the longitudinal and transverse characteristics of non-pharmacological management of neonatal and infant procedural pain and the trends and multiple branches associated with it, to offer new clues and ideas for the subsequent research on neonatal pain. It also aimed to determine the most effective pain relief methods tailored to specific clinical scenarios and to provide effective strategies for clinical medical staff to alleviate neonatal procedural pain, improve the comfort of neonates and infants, and minimize the short- and long-term effects of pain.

## 2. Methods

### 2.1. Data sources and literature search strategy

**Figure 1.**
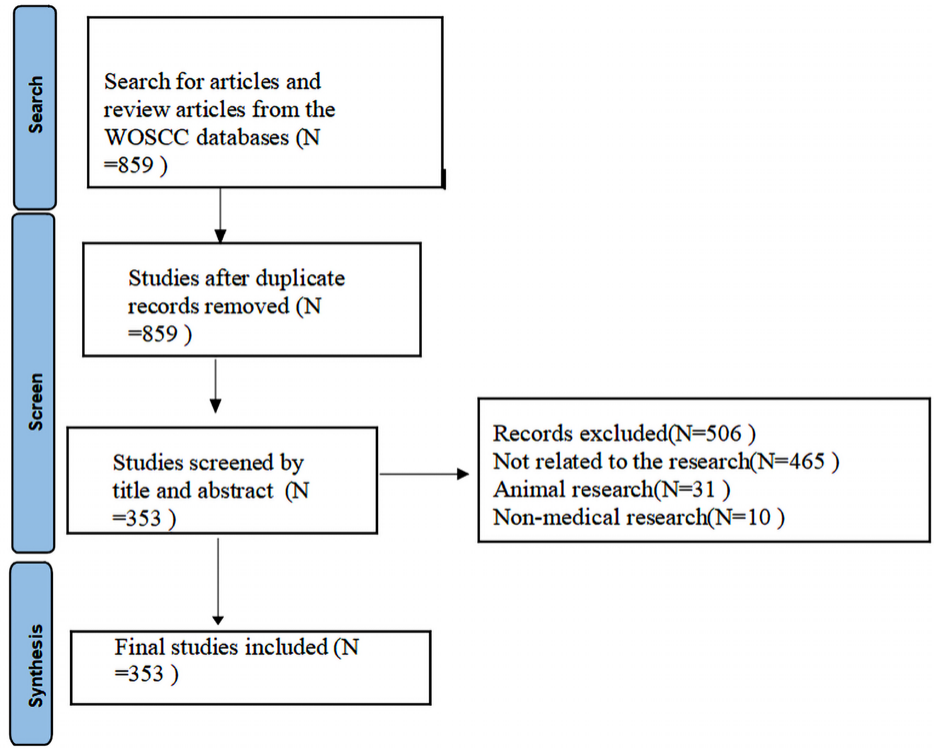
Article selection for bibliometric mapping analysis.

The Web of Science (WOS) database includes high-impact academic journals with comprehensive, international, and multidisciplinary coverage.^[24]^ Meanwhile, according to research findings, using CiteSpace for visual analysis has been observed to enhance the knowledge map effect of the Web of Science database.^[29]^ So, to improve the accuracy and comprehensiveness of the target article information, this research used the Web of Science Core Collection as the data source and selected the Science Citation Index Expanded and Social Sciences Citation Index as citation indexes. The following search terms were employed to retrieve literature from WoSCC: TS= (“non-pharmacological intervention” OR treatment OR mitigation OR reduction OR management OR intervention OR music OR “oral sucrose” OR “kangaroo care” OR “simulated intrauterine sound” OR “non-nutritive sucking” OR “skin to skin care” OR breastfeeding OR “heel warming” OR massage OR swaddling OR odor) AND TI = (neonatal pain OR infant pain OR newborn pain). We searched articles, review articles (Document Type) in English (Languages) published between 1989 and 2024 (Retrieval deadline: 2024.3.31). The literature screening was initiated with the first available article, Campos, 1989,^[30]^ on the Web of Science database. Following a thorough search, a total of 859 literature records were obtained. Subsequently, the content of each article (including the title and abstract of the paper) was manually reviewed to eliminate duplicate and irrelevant publications to ensure that the selected article involved the effect of non-drug therapy on neonatal pain. Finally, 353 articles were retained for bibliometric mapping analysis (Fig. 1) The summary of data source and selection is presented in Table 1.

**Table 1:** Summary of data source and selection

Category	Specific standard requirements
Research database	Web of Science core collection
Searching period	January 1985 to March 2024
Language	“English”
Search Keywords	#1TS= (“non-pharmacological intervention” OR treatment OR mitigation OR reduction OR management OR intervention OR music OR “oral sucrose” OR “kangaroo care” OR “simulated intrauterine sound” OR “non-nutritive sucking” OR “skin to skin care” OR breastfeeding OR “heel warming” OR massage OR swaddling OR odor) #2TI = (neonatal pain OR infant pain OR newborn pain) #1 AND #2 (In addition, it should be noted that the field tag “TS” refers to “Topic,” whereas the field tag “TI” means “Title.”)
Document Types	“Articles”, “Review articles”
Data extraction	Export with full records and cited references in plain text format
Sample size	353

**Figure 2.**
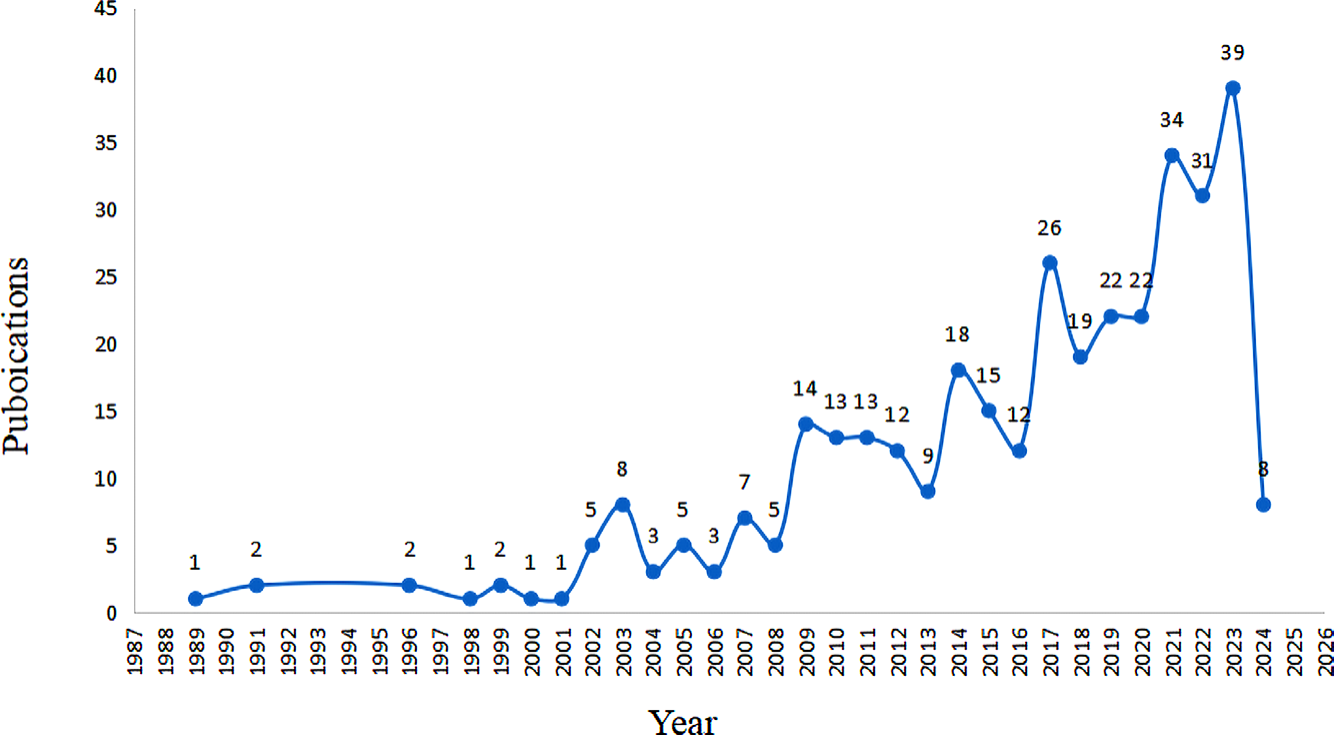
The number of publications on non-pharmacologic management of neonatal procedural pain research (1989–2024).

The number of works of literature on non-drug intervention for neonatal pain showed a continuous growth trend. The number of publications in 2023 was 4.875 times that in 2003, and it was not fully included in 2024, as shown in Figure 2.

### 2.2. Data Analysis

The VOSviewer and CiteSpace (v.6.2.3) analyzed all 353 documents. VOSviewer (a bibliometric software) is a program for constructing and viewing bibliometric maps developed by Van Eck and Waltman of the Center for Science and Technology Studies of Leiden University in the Netherlands in 2009. VOSviewer can carry out network analysis, co-occurrence analysis, citation analysis, literature coupling analysis, and co-citation analysis and visually display them with beautiful graphics to quickly lock the key research in the subject field.^[31]^ CiteSpace is an information visualization software developed by Professor Chen Chaomei of Drexel University, which offers advantages in the evaluative analysis of network visualizations.^[32]^

### 2.3. Ethical review

The data used in this study were from the Web of Science Core Collection (WOSCC) and did not involve patients or public contributions. Therefore, permission was not required from the ethics committee.

## 3. Results

### 3.1. Distribution of countries/regions, institutions, and authors

**Figure 3.**
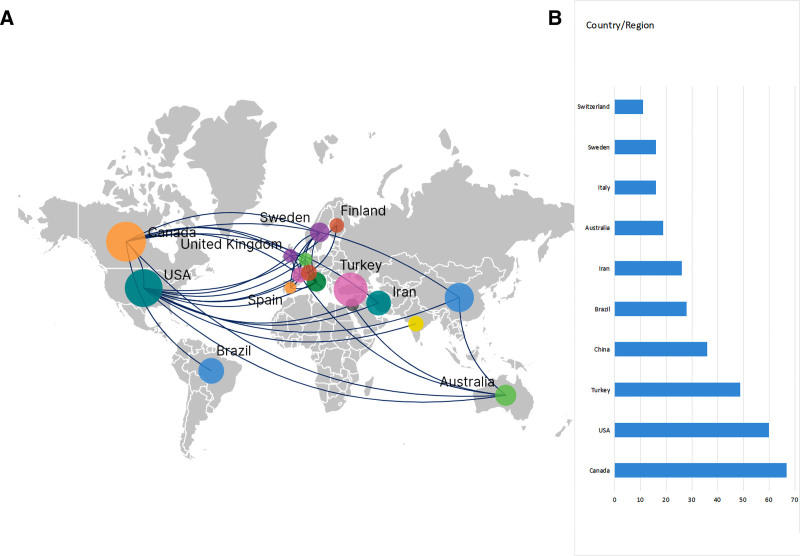
Country collaboration network (A) and top 10 countries (B) in the field of non-pharmacologic management on neonatal procedural pain (1989–2024).

The countries/regions, institutions, and authors included in the literature are visually analyzed by using VOSviewer and CiteSpace. From 1989 to 2024, a total of 51 countries, 548 institutions, and 1331 authors contributed to the publication of papers on non-pharmacological management of neonatal or infant pain. Figure 3 illustrates the theme’s national collaboration network (A) and the top 10 countries/regions (B). Each dot in the map represents a country/region; the larger the radius of the dot, the more papers are published. The connections between the dots denote the connection or cooperative relationship between the countries/regions. The closer the connection, the closer the cooperative relationship between the two. Canada has the largest number of neonatal/infant pain articles, with 67articles, followed by the USA with 60 articles, Turkey with 49 articles, China with 36 articles, Brazil with 28 articles, Iran with 26 articles, Australia with 19 articles, Italy and Sweden both with 16 articles, Switzerland with 11 articles. As shown in Figure 3, the USA cooperates most with other countries, including Australia, the UK, Canada, Italy, Japan, China, etc. Furthermore, Canada and Australia also cooperate more with other countries. In the relevant studies in the United States, newborns living in the NICU were the main subjects of their research. Their research specifically used methods such as kangaroo mother care, familiar odors, and skin-to-skin contact during medical procedures to help alleviate pain. Additionally, related research has explored the effects of early pain experiences on the neurodevelopment of premature infants.^[33–38]^

**Figure 4.**
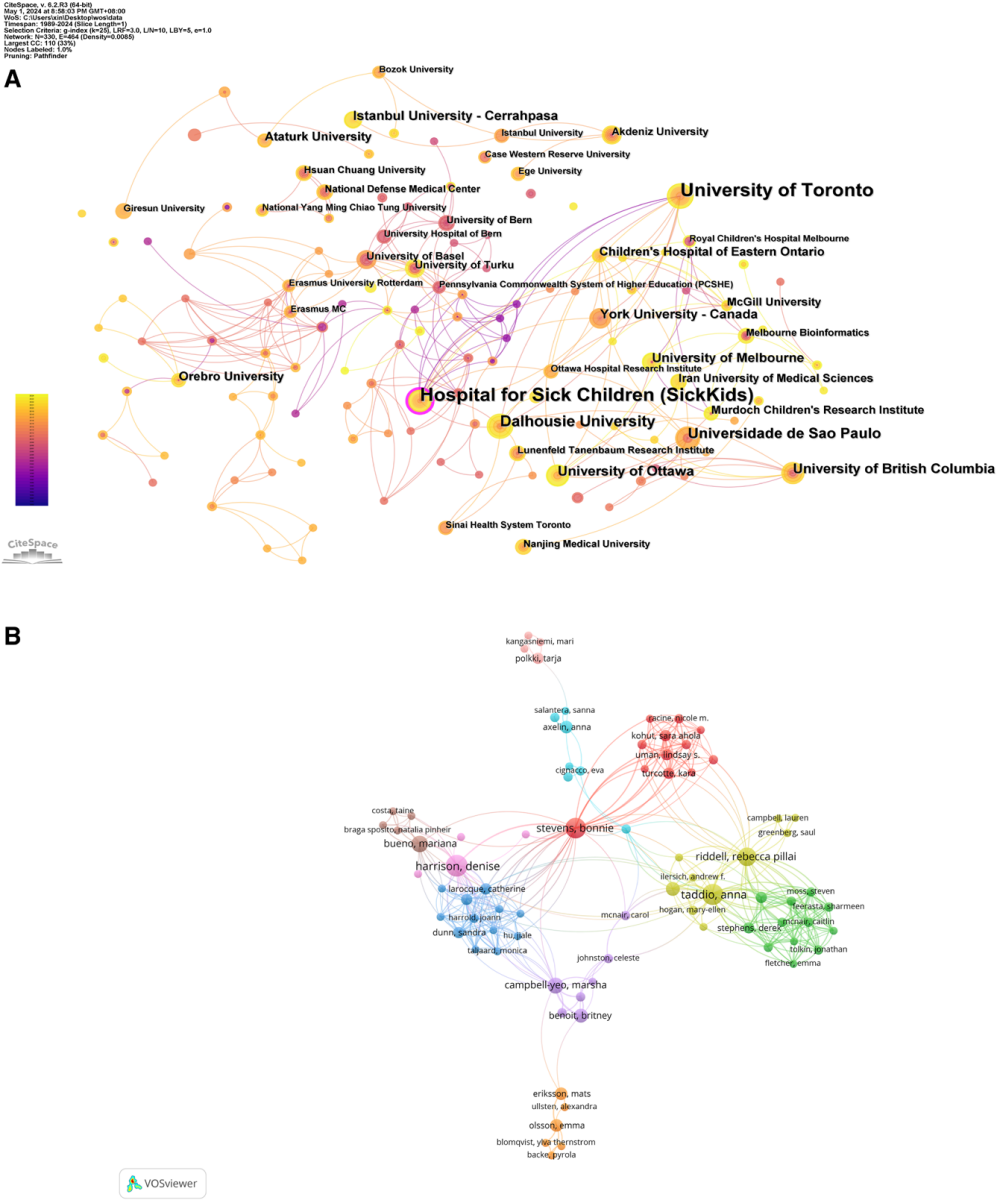
(A) Cooperation of institutions relating to non-pharmacologic management on neonatal procedural pain research publications (1989–2024). (B) Collaboration of authors on non-pharmacologic management of neonatal procedural pain (1989–2024). Different colors represent different clusters.

The institution module in CiteSpace is utilized to analyze the issuing institution of the document. The size of nodes represents the number of institutional documents. The larger the node, the more documents, and vice versa. Centrality measures the importance of nodes and reflects the importance of nodes. Figure 4A illustrates nodes = 330, and links = 561. Similarly, the top 10 institutional cooperation included the University of Toronto (30), Hospital for Sick Children (30), Dalhousie University (16), Universidade de Sao Paulo (14), University of British Columbia (11), University of Ottawa (11), York University (10), University of Melbourne (9), Istanbul University (9), Children’s Hospital of Eastern Ontario (8) (Table 2). Among these, the University of British Columbia’s (0.06) centrality and the University of Basel’s (0.06) centrality are the highest, followed by the Pennsylvania Commonwealth System of Higher Education (PCSHE) (0.05) and Hospital for Sick Children (0.04). The low centrality of all institutions indicates low inter-agency cooperation. Among a series of studies on this research theme by the University of Toronto, the most cited article is a continuously updated systematic review published in the Cochrane Database of Systematic Review by Rebecca R Pillar Ridell.^[39]^ In the latest updated review, it is pointed out that non-nutritive sucking, facilitated tucking, and swaddling may reduce pain behaviors in preterm-born neonates. Still, there was a lack of high-quality evidence to draw a definitive conclusion.^[40]^

**Table 2:** TOP 10 institutions with the most studies on non-pharmacologic management on neonatal procedural pain (1989–2024)

Count	Centrality	Year	Institution
30	0.03	2000	University of Toronto
30	0.04	2000	Hospital for Sick Children (SickKids)
16	0.02	2008	Dalhousie University
14	0.01	2004	Universidade de Sao Paulo
11	0.06	2004	University of British Columbia
11	0.01	2012	University of Ottawa
10	0.00	2011	York University - Canada
9	0.00	2003	University of Melbourne
9	0.00	2015	Istanbul University - Cerrahpasa
8	0.00	2012	Children’s Hospital of Eastern Ontario

Through an analysis of the scholars authoring the 353 retrieved papers, the most productive authors, including their connectionsto coauthor networks, are identified. For this analysis, the minimum number of papers published by an author is set at two. Overall, 179 authors met the criteria. The University of Ottawa’s Harrison Dennis is the most productive author, with 15 papers, followed by Tadio Anna (14) from the University of Toronto, Stevens Bonnie (13) from the Hospital for Sick Children, and Rebecca (11) from York University (Fig. 4B, Table 3). Cooperation among scholars has gradually become a trend that can promote the development of the discipline to a certain extent. Figure 4B shows that multiple cooperative sub-networks with the above-mentioned authors as the core have been formed in this field. There are strong academic ties between scholars, but there are collaborative relationships of varying strength outside the sub-network. Finding small groups of researchers in a field through a collaborative network of authors can help expand communication and collaboration with other researchers with different research directions.

**Table 3:** Top 10 authors with the most published studies on non-pharmacologic management of neonatal procedural pain (1989–2024)

Count	Author	Citations	Total link strength
15	Harrison, Denise	232	4044
14	Taddio, Anna	223	3607
13	Stevens, Bonnie	522	5553
11	Riddell, Rebecca Pillai	183	2462
9	Bueno,Mariana	160	2406
8	Campbell-yeo,Marsha	92	3470
7	Holsti,Liisa	131	1403
6	Benoit,Britney	59	2819
6	Shah,Vibhuti	59	1537
5	Eriksson,Mats	106	2577

### 3.2. Keyword analysis

**Figure 5.**
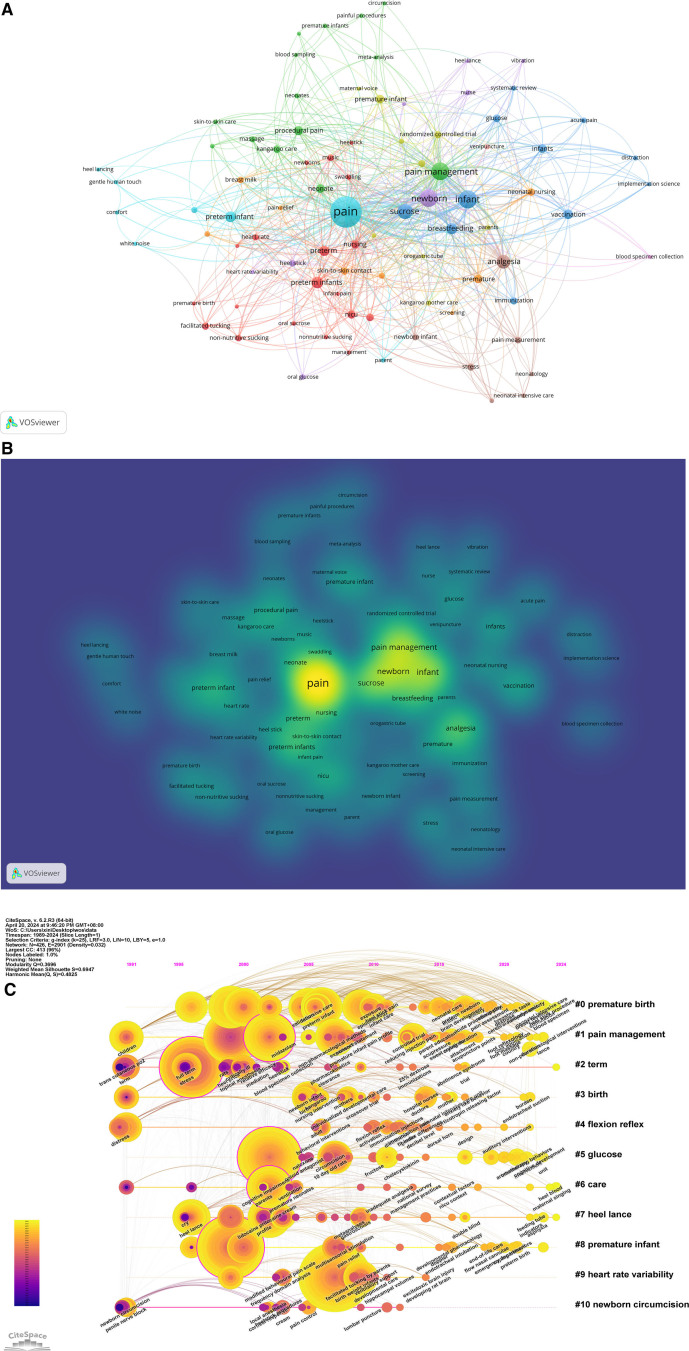
Keyword co-occurrence (A), keyword density map (B), and keyword timeline view (C) in the field of non-pharmacologic management on neonatal procedural pain (1989–2024).

Using CiteSpace and VOSviewer for keyword analysis can reveal research hotspots, frontiers, and trends. VOSviewer statistics show 948 keywords in 353 articles, of which 16 keywords appear more than 40 times, 33 appear more than 20 times, and 68 appear more than 10 times. Keyword co-occurrence, clustering, and density distribution are shown in Figure 5A and B. In Figure 5A each node represents one of the 948 keywords. The larger the circular node is, the more the keywords appear, and the more it can represent the hot spot in the field. The node connection represents the correlation strength, and the thicker the connection, the more times the two appear together in the same literature. In Figure 5B each point has a color that indicates the density of keywords at that point. Colors range from blue to green to yellow. The larger the number of keywords in the neighborhood of a point and the higher the weights of the neighboring keywords, the closer the color of the point is to yellow. We found that the keywords with the largest node, the highest density, and the highest frequency included pain, procedural pain, management, sucrose, analgesia, and preterm infant, which appeared 168 times, 115 times, 107 times, 102 times, 99 times, and 88 respectively, in line with our research topics. The pain procedures that infants may experience include subcutaneous or intramuscular injections, heel-prick, venipuncture, endotracheal suctioning pain, even tracheal intubation, lumbar puncture, supra-pubic bladder aspiration, and so on. Repeated procedural pain may lead to increased cortisol secretion and sleep-wake disorders in infants, leading to future neurobehavioral developmental disorders and harmful effects on cognitive behavioral development.^[41,42]^ Therefore, infant pain still needs to be adequately managed and intervened. It can be seen from the map of this study that the most used method is oral sucrose. Other keywords are also associated with non-pharmacological interventions for neonatal pain, such as non-nutritive sucking, skin-to-skin contact, facilitated tucking, kangaroo care, and breastfeeding.

Keywords are clustered using keyword-based log-likelihood ratio, and a timeline keyword graph is drawn to show each cluster’s difference in time and research progress. The clustering graph is evaluated based on two indexes: clustering modulus Q (Modularity Q) and clustering contour index S (Mean Silhouette). The S value is an index to measure the homogeneity within the entire cluster. The value of S determines how similar the members of a cluster are. Q > 0.3 indicates that the partition structure is significant, S > 0.5 indicates that the clustering is reasonable, and S > 0.7 indicates that the clustering is convincing.^[43]^

In our study, Modularity Q = 0.3696 > 0.3 and Mean Silhouette S = 0.6947 > 0.5 suggest clear and reliable keyword clustering. We conducted an log-likelihood ratio analysis of keywords and discovered 11 distinct natural clusters. These keyword-clustering tags represent the primary research hotspots and trends in this field. Figure 5C shows keyword clustering. Category 1(#0, #8) takes study subjects as the theme, including premature birth and premature infant; the second category (#7, #10) takes the sources of pain as the theme, including heel lance prick, newborn circumcision; the third category(#4, #9) clustering label is pain stimulation reactions, including flexion reflex, heart rate variability; the fourth category (#1, #5, #6) clustering label is pain intervention, including pain management, glucose, care; the other two clustering labels (#2, #3) are term and birth. Moreover, the largest cluster (#0) contains the most keywords, with the main keywords for premature birth, breastfeeding, and oral sucrose. Further consulting the relevant literature shows that an estimated 13.4 million newborn babies were born preterm (<37 weeks) in 2020 compared with 13.8 million in 2010 worldwide.^[44]^ Surviving preterm infants have undergone hundreds of diagnostic and therapeutic procedures, and their pain threshold is lower than that of full-term babies. The goals of managing pain in preterm infants include reducing the intensity and the behavioral and physiological consequences of pain, maximizing the ability of premature infants to cope with painful experiences, and providing interventions that provide the most benefit with low risk.^[45]^ Therefore, some non-drug interventions are used to alleviate the discomfort of preterm infants, including oral sucrose, breastfeeding, and so on.

**Figure 6.**
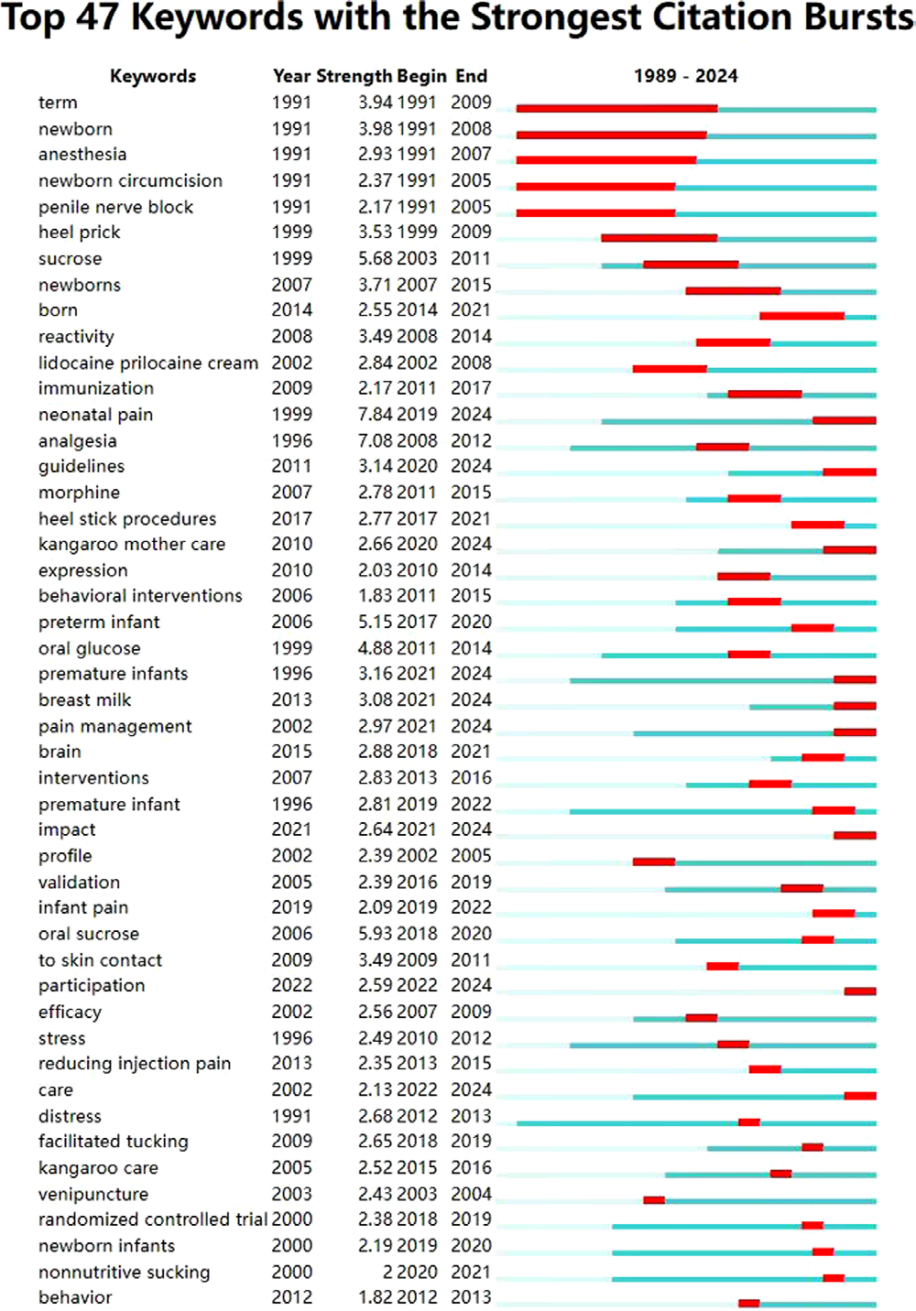
Top 47 burst keywords in the field of non-pharmacologic management on neonatal procedural pain (1989–2024).

Keyword emergence refers to the extraction of keywords with a high-frequency change rate in a certain period from many subject words by detecting the frequency of keywords. The minimum duration of occurrence in this study is one year. Figure 6 shows the top 47 emerging words in this field and the research hotspots in recent years. To a certain extent, it can predict the future development trend of non-drug intervention for neonatal pain. Burst keywords with high strength include “neonatal pain” (7.84, 2019–2024), “analgesia” (7.08, 2008–2012), “oral sucrose” (5.93, 2018–2020), “sucrose” (5.68, 2003–2011), “oral glucose” (4.88, 2011–2014), “heel prick” (3.53, 1999–2009) showing that they are hot topics of the research. Among them, the keywords “premature infants” (1996–2024) received the most sustained attention. The figure indicates that the most emerging keywords in research over the past 5 years are guidelines, non-nutritive sucking, breastfeeding, kangaroo mother care, facilitated tucking, premature infant, pain management, impact, and participation. These words represent the current research hotspots and suggest that future research will likely focus on these keywords.

### 3.3. Highly cited papers analysis

**Figure 7.**
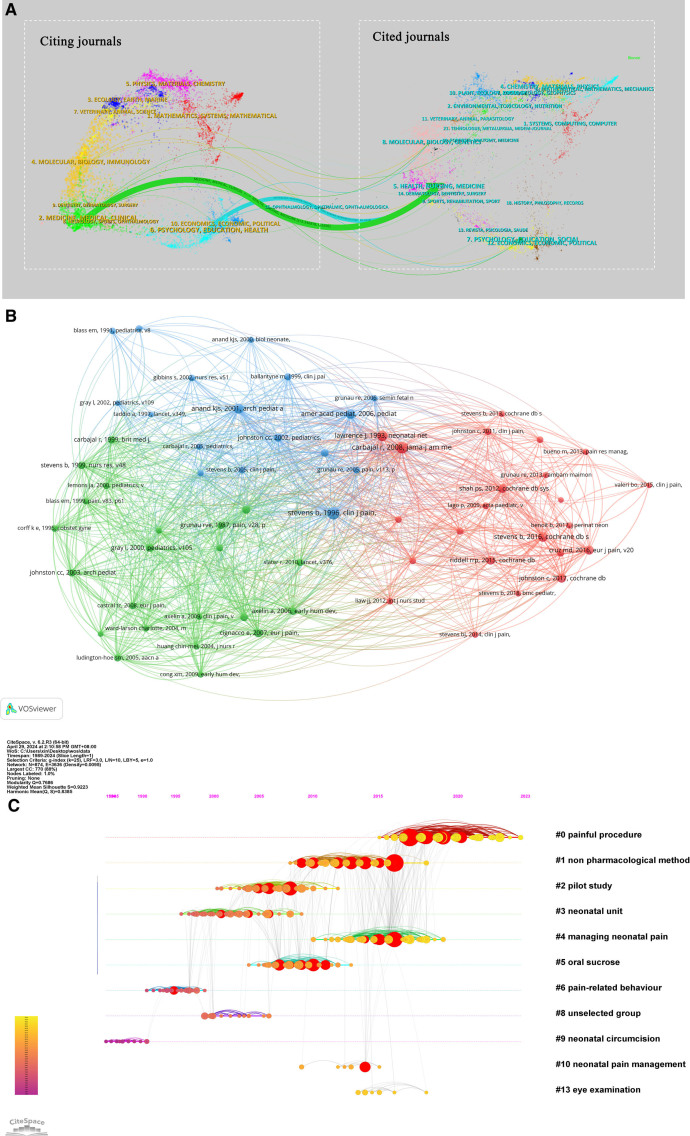
(A) Dual map of journals published on non-pharmacologic management of neonatal procedural pain (1989–2024). The cited journal is on the right, the citing journal is on the left, and the straight path represents the citation relationship. (B) Co-cited references in the field of non-pharmacologic management on neonatal procedural pain (1989–2024). (C) Timeline view of co-cited papers of non-pharmacologic management on neonatal procedural pain with cluster labels (1989–2024).

The analysis of citations is considered an important part of bibliometrics research. It is a collection of all previous literature and the basis and evidence of the research frontier of a discipline. The distribution of links between journals is shown in a dual map overlay of journals, with the citing journals on the left and the cited journals on the right. The relationships described are represented by the colored lines connecting them. These labels represent the topics covered by the journals. Two main reference paths are identified-green and cyan. The green path includes studies published in Medical/Clinical/Health/Nursing journals that are cited by Health/Nursing/Medical journals. The cyan pathway represents studies published in Molecular/Psychological/Educational/Health journals that are cited by Molecular/Biological/Educational journals (Fig. 7A).

VOSviewer analyzes a total of 6758 references, and the minimum citation frequency is set to 20. The co-citation correlation analysis and visual map are performed on the 62 references included, as shown in Figure 7B. VOSviewer software divides the above references into 3 clusters, each of which is represented by different colors. Red is Cluster 1, green is Cluster 2, and blue is Cluster 3. The larger the circular node is, the more cited references it represents. Among them, the largest node represents the most cited article, which was published Clinical Journal of Pain in 1996. It showed that the lack of pain assessment for premature infants was a long-standing problem, and The Premature Infant Pain Profile (PIPP) has content and structural validity.^[46]^

As shown in the Figure 7C, nodes = 874, links = 3636, S = 0.9223. there are 11 categories, including #0 painful procedure, #1non pharmacological method, #2 pilot study, #3 neonatal unit, #4 managing neonatal pain, #5 oral sucrose, #6 pain-related behavior, # 7 unselected group, #8 neonatal circumcision, #9 neonatal pain management, #10 eye examination. To better understand highly co-cited papers, we read the top 10 papers promoting this field’s development, including articles and reviews.

The top count co-cited reference is the article published in the Cochrane Database of Systematic Reviews in 2017 entitled “Skin-to-skin care for procedural pain in neonates,” which shows that skin-to-skin contact (SSC) is effective by combining pain indicators and physiological behavior indicators. The synergistic effect of this intervention and other interventions will be explored in the future.^[47]^ In addition, it is followed by a systematic review published in the same journal in 2016 entitled “Sucrose for analgesia in newborn infants undergoing painful procedures,” which shows that sucrose could effectively reduce procedural pain from single events such as heel lance, venipuncture, and intramuscular injection in both preterm and full-term infants. This intervention has no serious side effects or harm. However, further research is needed to determine the lowest effective dose of sucrose during a single pain session and the effects of repeated administration of sucrose on immediate (pain intensity) and long-term (neurodevelopmental) outcomes.^[20]^ There were also highly co-cited articles, such as one published in the European Journal Of Pain in 2016 with the title “Epidemiology of painful procedures performed in neonates: A systematic review of observational studies”^[2]^ and a 2008 article published in JAMA-Journal of the American Medical Association with the title of “Epidemiology and treatment of painful procedures in neonates in intensive care units ”^[48]^ and so on (Table 4).

**Table 4:** Top 10 co-cited references concerning non-pharmacologic management on neonatal procedural pain (1989–2024)

Rank	Author	Title	Co-cited count	Journal	Year	IF (2023)
1	Celeste Johnson	Skin-to-skin care for procedural pain in neonates	32	Cochrane Database of Systematic Reviews	2017	8.8
2	Bonnie Stevens	Sucrose for analgesia in newborn infants undergoing painful procedures	30	Cochrane Database of Systematic Reviews	2016	8.8
3	Cruz, MD	Epidemiology of painful procedures performed in neonates: a systematic review of observational studies	22	European Journal of Pain	2016	3.5
4	Carbajal, Ricardo	Epidemiology and treatment of painful procedures in neonates in intensive care units	20	Jama – Journal of The American Medical Association	2008	63.1
5	Mangat, Avneet k	A review of non-pharmacological treatments for pain management in newborn infants	19	MDPI	2018	2.0
6	Bucsea, Oana	Non-pharmacological pain management in the neonatal intensive care unit: Managing neonatal pain without drugs	18	Seminars In Fetal & Neonatal Medicine	2019	2.9
7	Bonnie Stevens	Sucrose for analgesia in newborn infants undergoing painful procedures	18	Cochrane Database of Systematic Reviews	2010	8.8
8	Bonnie Stevens	The minimally effective dose of sucrose for procedural pain relief in neonates: a randomized controlled trial	17	BMC Pediatrics	2018	2.0
9	Celeste Johnson	Skin-to-skin care for procedural pain in neonates	17	Cochrane Database of Systematic Reviews	2014	8.8
10	Bonnie Stevens	Sucrose for analgesia in newborn infants undergoing painful procedures	16	Cochrane Database of Systematic Reviews	2013	8.8

## 4. Discussion

This study focused on non-pharmacological interventions for neonatal procedural pain and reported this research topic’s quantitative and qualitative bibliometric analysis for the first time, covering 353 research articles from 1989 to 2024 from the WOSCC. Moreover, through the visual analysis method, this study deeply explored the hot changes in non-drug management of neonatal procedural pain and its future development trend, provided a new perspective for related research in the world, and aroused the attention of clinical medical personnel on neonatal/infant procedural pain experiences. This may further advance the field of pediatric pain.

The analysis of countries/regions, institutions, and authors shows that Canada has published more papers and played a “bridge” role in the field of non-pharmacological strategies for neonatal/pain. In terms of international cooperation, the United States demonstrated strong collaboration and the highest centrality with other countries. The above findings confirm the US’s and Canada’s critical contributions and leading position in non-pharmacologic management on neonatal procedural pain research, which may result from the United States’ and Canada’s national economic conditions and high medical investment levels. This field will benefit from extensive international cooperation, which will improve the overall standard of research. But in general, the cooperation is localized, reflecting that there is no unified model of non-drug intervention for neonatal/infant pain among countries.

Furthermore, in analyzing the contributions made to the research, it was found that universities have played a significant role. Some of the prominent universities that have contributed to the research include the University of Toronto, Dalhousie University, Universidade de Sao Paulo, University of British Columbia, University of Ottawa, York University, and more. These contributions have greatly facilitated the advancement and development of the research.

Based on the author’s analysis, Professor Harrison Dennis from the University of Ottawa is the most prolific scholar in the field of neonatal/infant procedural pain. Research areas include infant pain assessment tools^[49–51]^ and interventions such as sweet solutions,^[52,53]^ breastfeeding,^[54]^ multidimensional knowledge translation strategies^[55]^ to improve procedural pain in newborns, and so on. In addition, this study also shows strengthening exchanges and cooperation between researchers around the world will contribute to the development of non-drug interventions for neonatal/infant procedural pain.

Keywords reflect the core content of a study, and co-occurrence analysis can identify high-frequency keywords that appear in different studies to help researchers quickly grasp research hotspots.^[56]^ For keyword co-occurrence, the keywords with higher frequency were closely related to the source of pain, pain management, analgesic modality, and the main study population of neonatal pain. Emergent keywords indicated emerging trends and research frontiers. Through keyword hotspot analysis, the keyword hotspots of neonatal pain focused on preterm infants, procedural pain, oral sucrose, and heel stick. Preterm infants admitted to hospitals are often exposed to many painful interventions, including venous blood collection.^[57]^ Heel stick is the most common painful procedure for preterm infants in neonatal intensive care units.^[34]^ Sucrose has been widely used as a standard therapy for minor procedural pain.^[58,59]^ This will provide valuable options for clinicians to minimize procedural pain and discomfort in newborns and infants.

We found that papers on non-drug interventions for neonatal pain were mostly published in influential journals like Pediatrics, Pain, Cochrane Database of Systematic Reviews, JAMA-Journal of the American Medical Association, etc. The publication of these journal articles on this topic demonstrates that neonatal/infant pain remains a major issue for pediatrics and pain research. From 1989 to 2024, the top 15 co-cited literature proves that neonatal pain research mainly focuses on pain assessment, intervention measures, and evaluation of intervention effects. etc.

The study has some limitations. The WOSCC database is regarded as the most crucial data source for bibliometric analysis, and it was the only database used in this study. However, it’s possible that some studies were not included. Moreover, the analysis only considered articles, reviews, and publications in the English language. This could have resulted in some bias.

## 5. Conclusions

This study used the method of bibliometrics to conduct a thorough review of non-drug interventions related to neonatal procedural pain. The analysis covers many aspects, such as the number of publications, distribution of countries, institutions, authors, keywords, highly-cited papers, and co-citation papers, etc. The results indicate that researchers worldwide are increasingly focusing on neonatal procedural pain. Strengthening the cooperation between countries and institutions is conducive to promoting research in the field of neonatal pain. The institutions with the most international collaboration are the University of British Columbia and the University of Basel, with the USA being the top collaborator with other countries. Importantly, through this study, we analyzed the research foundation, current trends, and future directions for the non-pharmacological management of neonatal procedural pain. These results facilitate new researchers to quickly understand the authoritative institutions, authors, literature, and cutting-edge trends in the field, as well as help clinical staff quickly master methods for relieving procedural pain in neonates. In short, to promote the development of neonatal/infant pain management, it is important to study further, explore the best prevention and intervention measures, and conduct more convincing, high-quality research.

## Author contributions

**Conceptualization:** Xin Chen.

**Data curation:** Anqi Xiong.

**Methodology:** Ruoyu Li, Biru Luo.

**Resources:** Xin Chen.

**Software:** Xin Chen.

**Writing – original draft:** Xin Chen.

**Writing – review & editing:** Biru Luo.
